# Sex-specific associations of different risk behaviors with socio-demographic, health status and lifestyle factors

**DOI:** 10.1192/j.eurpsy.2023.825

**Published:** 2023-07-19

**Authors:** J. Kose, P. Duquenne, S. Hercberg, P. Galan, M. Touvier, L. K. Fezeu, A. A. Valentina

**Affiliations:** Equipe de Recherche en Epidémiologie Nutritionnelle-Université Sorbonne Paris Nord, Bobigny, France

## Abstract

**Introduction:**

Knowledge about concurrent substance use and other risk behaviors - as well as their determinants - in the general population is insufficient.

**Objectives:**

To investigated socio-demographic, health status, and lifestyle determinants of habit-forming risk behaviors among French men and women.

**Methods:**

We analyzed data collected in 2021–2022 from 32,622 participants (74.5% female; mean age=57.9±14.2 years) of the NutriNet-Santé web-cohort who had completed the Alcohol Use Disorders Identification Test, the 12-item Cigarette Dependence Scale, the modified Yale Food Addiction Scale 2.0, and the Internet Addiction Test. Using established cutoff values, participants were first split into 2 groups (Yes/No) for each risk behavior (alcohol use disorders, nicotine dependence, food addiction, and Internet addiction) and then placed into 3 groups (no risk behavior, 1 risk behavior (reference), and ≥2 risk behaviors) before fitting polytomous logistic regression models according to sex.

**Results:**

Younger age (Odds Ratio: OR_male_=2.07; OR_female_=2.04), self-perceived poor health (OR_male_=2.06; OR_female_=1.61), having obesity (OR=1.56; OR_female_=1.30), lack of affection during childhood (OR_male_=1.56; OR_female_=1.39), and a lifetime prevalence or medication use for a mental disorder (OR_male_=1.73; OR_female_=1.38) were significantly associated with having ≥2 versus 1 habit-forming risk behavior in both sexes (all *p*<0.05). Results for experiencing current financial difficulties (OR_female_=1.34), self-perceived poor dietary quality (OR_female_=3.23), being underweight (OR_female_=1.58) and overweight (OR_female_=1.30) were significant only in females (all *p*<0.05). The same factors plus current e-cigarette use (OR_male_=0.54; OR_female_=0.77) in both sexes, having a higher educational attainment (OR_female_=0.75), being physically active at work (OR_female_=0.78) in females were inversely associated with having none versus 1 risk behavior (all *p*<0.05).

**Conclusions:**

To our knowledge, this is the first study to explore determinants of concurrent habit-forming risk behaviors among men and women in a large, population-based study. The findings could serve as impetus for future research in this domain and ultimately help guide addiction prevention efforts.

**Disclosure of Interest:**

None Declared

